# Herpes Virus Pseudotumor in a Patient with HIV Immunosuppression

**DOI:** 10.1155/2022/3109331

**Published:** 2022-07-07

**Authors:** Daniel York, Pavan Patel, Smera Saikumar

**Affiliations:** ^1^Colquitt Regional Medical Center, Sterling Center Women's Health, Moultrie GA 31768, USA; ^2^Philadelphia College of Osteopathic Medicine, Moultrie, GA 31768, USA

## Abstract

**Background:**

In rare cases, HSV infections can present as pseudotumors that are often mistaken as malignancies in patients with an uncontrolled HIV infection. Herpes simplex virus type 2 (HSV-2) infection rates range from 60% to 90% in individuals coinfected with HIV. *Case Presentation*. A 48-year-old patient presented with a large fungating mass near her right inferior vulva with a hardness of surrounding tissues. The mass was 4 cm × 3 cm in size and was excised in the operating room. The pathology was negative for malignancy; however, it showed lymphoplasmacytic proliferation with immunostaining positive for HSV virus.

**Conclusion:**

Atypical HSV pseudotumors should be considered in the differential diagnosis for an immunosuppressed patient who presents with a genital mass lesion.

## 1. Introduction

Herpes genitalis, one of the most common sexually transmitted infections (STIs), is a significant source of morbidity in patients infected with human immunodeficiency virus (HIV) [1]. Herpes simplex virus type 2 (HSV-2) infection rates in HIV-infected patients range from 60% to 90% in different regions of the world [2]. While most HSV-2 infections are subclinical, the clinical manifestations of symptomatic patients include recurrent orolabial lesions, painful genital or anal ulcerations, tender lymphadenopathy, and dysuria [3]. Systemic symptoms such as fever, headache, and malaise may also occur [4]. Although ulcerations are the most common presentations in those with HSV, atypical nodules simulating neoplasms or hypertrophic lesions may occur in those with HIV [5].

The clinical manifestations of HSV infection in immunocompromised patients, such as those positive for HIV, are more likely to be severe, disseminated, atypical, or chronic HSV infection [6]. The risk of developing an HSV infection is also increased in HIV-immunodeficient patients. Recurrent anogenital lesions, shedding, and disease are also more frequent and severe for those that are HIV-immunodeficient. HSV infection causes an increase in HIV replication and in the risk of HIV transmission [7]. Due to this, patients with HIV should be frequently tested for HSV-2 infections and offered education and treatment options to reduce the severity of infection [8].

## 2. Case Presentation

**Figure 1:**
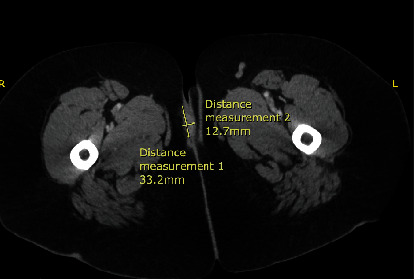
CT scan of the fungating mass with surrounding edema near the right vulvar region, near the groin crease.

PP is a 48-year-old female with a past medical history significant for HIV who presented to the emergency department for evaluation of urinary hesitancy and pelvic pain. She also complained of pelvis discomfort due to a mass on the right side of her vulva, which had been present for a week. Upon examination, the mass was noted to be large and fungating to the right inferior vulva with hardness within the vaginal introitus and periurethral space. Bedside attempts were made for catheter placement, but the insertion was unsuccessful due to the suspected tumor involvement into the periurethral space. Labs prior to the procedure indicated a CD4 count of 104 cells/*μ*L with a viral RNA load of HIV virus 211 copies/mL. The CT scan showed mild induration of the right superficial vulva of 3.3 cm in AP dimension by 1.3 cm in the transverse direction (Figure 1). The decision was made to proceed to the OR for suprapubic catheter placement as well as vulvar biopsies with frozen sections.

**Figure 2:**
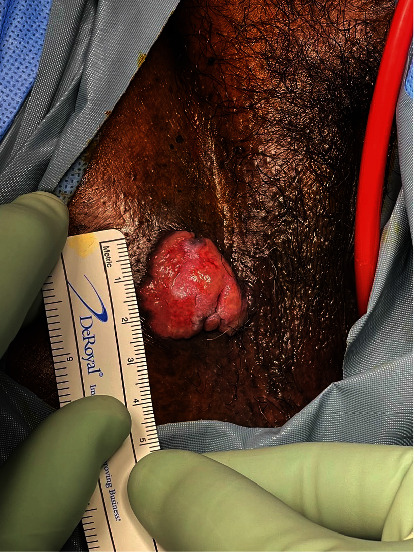
Raised fungating mass within the right inferior vulva measuring approximately 4 cm × 3 cm.

**Figure 3:**
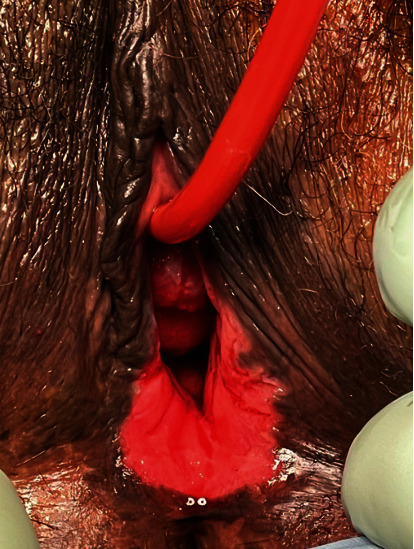
Suspected tumor at the vaginal opening and a suspected tumor covering the urethral meatus.

The patient was taken to the operating room, where general anesthesia was attained. Examination of the patient's pelvis revealed a fungating mass within the right inferior vulva that measured approximately 4 cm × 3 cm (Figure 2). It was also raised off the skin bed at approximately 1 cm. On palpation, there was noted to be a suspected tumor present at the vaginal opening, as well as the hardness of the surrounding tissues (Figure 3). Likewise, in the periurethral area, there was a suspected tumor present that was covering the urethral meatus, making it difficult for the patient to urinate. Despite this, urology was able to dilate the urethra, and a 16 French catheter was placed. Biopsies were taken of both at the 4 o'clock position in the periurethral area and vaginal introitus on the left inferior aspect. An additional biopsy was taken at the side of the 4 cm right inferior vulvar mass, which was nearing the groin crease.

The pathology report of the right inferior vulvar mass was negative for malignancy, CMV, and Treponema infection. However, it showed ulceration, exuberant lymphoplasmacytic proliferation, and pseudoepitheliomatous hyperplasia with immunostain positive for the HSV virus. The biopsies were negative for CD138, MUM-1, CD20, D3, CD56, Cyclin D1, and kappa and lambda, effectively ruling out neoplastic processes. The patient started on antiviral medications after her diagnoses of HSV. She reported improvement in her symptoms at her post-op appointment, and her sutures were removed at this time. She was advised to continue follow-up with her gynecologist and infectious disease doctor.

## 3. Discussion

Herein, we have discussed a case report of herpes genitalis in a patient with HIV immunodeficiency. Herpes genitalis is one of the most common STIs and is a major source of morbidity in those with HIV-induced immunodeficiency [1]. Viral replication occurs in epithelial tissues; however, it maintains dormancy in dorsal sensory neurons, thereby causing recurrent localized lesions [4]. Microscopically, HSV lesions are defined by the presence of dense lymphoid infiltrates with atypical lymphocytes [4].

Further investigation shows that healed lesions from HSV-2 infections also present an increased risk of acquiring HIV [9]. During acute infection, CD4 and CD8 cells infiltrate the epidermis and the dermis of the ulcer site to eliminate the HSV-2 virus particle. The average number of cells present at the ulcer site for CD4 and CD8 is 655 and 618, respectively, while in uninfected skin, it is 68 and 55, respectively [9]. When the skin lesion heals, the relatively increased number of the CD4 and CD8 cells becomes trapped in the dermis and epidermis. Furthermore, these entrapped CD4 cells showed receptors for CCR5 and CXCR4. On average, 55% of the lesions showed CCR5 receptors, which are six times greater than T-cells present in peripheral blood. This increased CCR5 receptor effectively became a target environment for the CCR5 topic HIV virus [9].

Due to the significant interaction between HSV and HIV, more severe and chronic HSV infections are seen in HIV-induced immunodeficient patients. HSV-2 infections have been shown to increase the rate of acquiring HIV threefold [2]. Hence, the early diagnosis of HSV-2 and early treatment can prevent possible transmission of the virus to others and lower the risk of acquiring HIV in those patients. For individuals who are negative for HIV and HSV that are engaging in a sexual relationship with a partner who is positive for both HSV and HIV, prophylactic treatment with antiviral medications can reduce the risk of contracting HSV and HIV infections [10]. In patients with HIV and AIDS, studies suggest that an aggressive treatment route with HAART therapy along with HSV antiviral medications can provide an improved survival benefit [5].

Although genital and perianal herpetic lesions are common in those with HIV, unusual tumor-like masses or pseudotumors simulating neoplasia may occur in those with HIV immunodeficiencies [5]. These atypical presentations can lead to increased misdiagnoses and delayed treatments in patients. Therefore, it is critical for physicians to be able to identify these unusual presentations to avoid such mistakes [5]. Close follow-ups and routine testing for HSV-2 should also be conducted in patients with HIV to reduce recurrences and disseminated infections.

## 4. Conclusion

HSV pseudotumors are present in patients with uncontrolled HIV infection. Any patient that presents with a tumor-like mass in their perineum should be screened for HSV lesions while also exploring the diagnosis of a neoplastic process. Awareness of atypical presentations of HSV lesions in patients with HIV immunodeficiencies can reduce misdiagnoses and treatment delays.

## Additional Points

*Précis*. Patients with perineal tumor-like masses should be screened for HSV pseudotumor lesions while also exploring the diagnoses of a neoplastic process.

## References

[1] Lolis M. S., González L., Cohen P. J., Schwartz R. A. (2008). Drug-resistant herpes simplex virus in HIV infected patients. *Acta dermatovenerologica Croatica: ADC*.

[2] Looker K. J., Elmes J. A., Gottlieb S. L. (2017). Effect of HSV-2 infection on subsequent HIV acquisition: an updated systematic review and meta-analysis. *The Lancet Infectious Diseases*.

[3] Nag S., Sarkar S., Chattopadhyay D., Bhattacharya S., Biswas R., SenGupta M. (2015). Seroprevalence of herpes simplex virus infection in HIV co-infected individuals in eastern India with risk factor analysis. *Advances in Virology*.

[4] Mathew J., Sapra A. (2022). Herpes Simplex Type 2. *StatPearls*.

[5] Mosunjac M., Park J., Wang W. (2009). Genital and perianal herpes simplex simulating neoplasia in patients with AIDS. *AIDS Patient Care and STDs*.

[6] Dinotta F., De Pasquale R., Nasca M. R., Tedeschi A., Micali G. (2009). Disseminated herpes simplex infection in a HIV+ patient. *Giornale Italiano Di Dermatologia E Venereologia: Organo Ufficiale, Societa Italiana Di Dermatologia E Sifilografia*.

[7] Aoki F. Y. (2001). Management of genital herpes in HIV-infected patients. *Herpes: The Journal of the IHMF*.

[8] Strick L. B., Wald A., Celum C. (2006). Management of herpes simplex virus type 2 infection in HIV type 1-infected persons. *Clinical infectious diseases: an official publication of the Infectious Diseases Society of America*.

[9] Zhu J., Hladik F., Woodward A. (2009). Persistence of HIV-1 receptor–positive cells after HSV-2 reactivation is a potential mechanism for increased HIV-1 acquisition. *Nature Medicine*.

[10] Ng B. E., Rutherford G. W., White A. B., Moore D. M., Cochrane HIV/AIDS Group (2018). Antiviral therapy for genital herpes for prevention of HIV transmission. *The Cochrane Database of Systematic Reviews*.

